# *Orientia tsutsugamushi* Alters Mitochondrial Function and Selectively Associates with VDAC

**DOI:** 10.3390/pathogens15040372

**Published:** 2026-03-31

**Authors:** Savannah E. Sanchez, Travis J. Chiarelli, John S. Billingsley, Richard T. Marconi, Jason A. Carlyon

**Affiliations:** Department of Microbiology and Immunology, Virginia Commonwealth University Medical Center, School of Medicine, Richmond, VA 23298, USA; ssanch1@midwestern.edu (S.E.S.); travis.chiarelli@vcuhealth.org (T.J.C.); billingdleyjs@vcu.edu (J.S.B.); richard.marconi@vcuhealth.org (R.T.M.)

**Keywords:** *Orientia tsutsugamushi*, scrub typhus, obligate intracellular bacterium, mitochondrial dysfunction, host–pathogen interactions, voltage-dependent anion channel (VDAC)

## Abstract

*Orientia tsutsugamushi* is an obligate intracellular alphaproteobacterium and the causative agent of the potentially fatal rickettsiosis, scrub typhus. During infection, *O. tsutsugamushi* replicates exclusively in the eukaryotic cytosol near mitochondria and alters host metabolic pathways governed by mitochondria. We report that *O. tsutsugamushi* induces mitochondrial enzymatic impairment and structural abnormalities without altering mitochondrial abundance or the levels of proteins that maintain mitochondrial homeostasis. Confocal and structured illumination microscopy revealed a selective spatial association between *O. tsutsugamushi* and the mitochondrial membrane protein, voltage-dependent anion channel (VDAC) but not other mitochondrial proteins. Immunosignal for VDAC paralogs 1 and 3 colocalized with cytosolic *O. tsutsugamushi* organisms whereas VDAC2 did not. Additionally, the antibody specific for VDAC1 and VDAC3 detected proteins of the expected sizes in *Orientia* membrane fractions. These findings indicate that *O. tsutsugamushi* negatively impacts mitochondrial function without overt organelle loss and selectively associates with VDAC1/VDAC3.

## 1. Introduction

*Orientia tsutsugamushi*, a causative agent of scrub typhus, is a mite-borne obligate intracellular bacterium. While most human infections with *O. tsutsugamushi* result in acute febrile illness, severe complications including myocarditis, encephalitis, and systemic vascular collapse can develop [1,2,3,4]. Without appropriate treatment, case fatality rates can reach 30% or higher [5]. Endemic transmission of scrub typhus primarily occurs in the Asia-Pacific, where it is considered the leading cause of febrile illness after malaria in certain locations [6,7,8]. However, the emergence of non-travel-related cases in the Middle East, Africa, and South America, together with the detection of *Orientia* in mites in North Carolina, USA, and the estimated one billion people worldwide to be at risk for infection, collectively underscore that scrub typhus is a global public health concern [9,10,11,12,13].

*O. tsutsugamushi* causes systemic disease by invading leukocytes, endothelial cells, and cardiomyocytes, where the bacterium replicates exclusively in the host cell cytosol [14,15,16,17,18]. Following invasion and escape from the endolysosomal pathway, the pathogen utilizes dynein-dependent motility to traverse to the microtubule-organizing center (MTOC) where bacterial proliferation initiates to form a microcolony [19,20,21,22]. Localized perinuclear proliferation is postulated to be strategic, offering access to host-derived resources and protection from degradation. Mitochondria also preferentially localize to the MTOC and similarly accomplish this by traveling along microtubules [23,24]. During infection, *Orientia* and mitochondria are often observed in close proximity to each other, with heavy infection leading to mitochondrial swelling [15,17,25]. Moreover, metabolic processes regulated by mitochondria including amino acid and fatty acid metabolism and the tricarboxylic acid (TCA) cycle are significantly reduced in *O. tsutsugamushi*-infected cells, which coincides with dampened ATP yields [26].

*Orientia* globally modulates host cellular processes and their governing organelles likely to promote a cellular environment that is permissive for its intracellular proliferation. This includes dysregulating transcription and translation [27,28,29], and attenuating innate and adaptive immunity [30,31,32,33]. Given that mitochondria command global eukaryotic functions including metabolism, apoptosis, and autonomous immunity [34], an interaction between *O. tsutsugamushi* and mitochondria would be consistent with known pathogen behavior. Additionally, mitochondrial homeostasis is a dynamic process, whereby the organelle’s functional roles are coordinated with quality control measures by regulatory proteins [34]. For instance, the mitochondrial voltage-dependent anion channel (VDAC) is implicated in coordinating activation of mitochondrial programmed cell death with mitochondrial mediated-metabolism, redox signaling, and inter-organelle communication [35]. Notably, *O. tsutsugamushi* delays apoptosis through various mechanisms [29,36,37,38,39,40,41].

Despite the circumstantial evidence indirectly linking *O. tsutsugamushi* and mitochondria, a functional relationship between the two has yet to be systematically investigated. Here, we assessed the effect of *O. tsutsugamushi* on host mitochondrial health and integrity throughout infection in two host cell types. Our results indicate that *O. tsutsugamushi* adversely affects mitochondrial function and selectively localizes with VDAC paralogs 1 and 3 but not VDAC2 or other mitochondrial proteins.

## 2. Materials and Methods

### 2.1. Cell Culture Maintenance and Media

HeLa human cervical epithelial cells (CCL-2; American Type Culture Collection (ATCC, Manassas, VA, USA)) and EA.hy926 human somatic cell hybrid endothelial cells (CRL-2922; ATCC, Manassas, VA, USA) were cultured in complete RPMI 1640 medium containing 11 mM glucose (Gibco/Thermo Fisher Scientific, Waltham, MA, USA) supplemented with GlutaMAX (Gibco/Thermo Fisher Scientific, Waltham, MA, USA), 15 mM HEPES (Gibco/Thermo Fisher Scientific, Waltham, MA, USA), and 5% (vol/vol) heat inactivated fetal bovine serum (hiFBS; Gemini Bio-Products, West Sacramento, CA, USA). Both cell lines were maintained at 37 °C in a humidified incubator with 5% CO_2_.

### 2.2. O. tsutsugamushi Cultivation and Infection

*O. tsutsugamushi* str. Ikeda (NC_010793.1), which was originally isolated from a scrub typhus patient in Japan [42], was propagated in HeLa cells cultured in complete RPMI 1640 medium and incubated at 37 °C in a humidified incubator with 5% CO_2_. To obtain *O. tsutsugamushi* for experimental use or re-propagation, bacteria were isolated from infected HeLa cells at 3 d post-infection via mechanical lysis. Briefly, media from individual 75-cm^2^ flasks containing *O. tsutsugamushi*-infected HeLa cells was discarded and cells washed once with phosphate-buffered saline (PBS; 2.7 mM KCl, 1.8 mM KH_2_PO_4_, 10 mM NaH_2_PO_4_, 137 mM NaCl, pH 7.4) before being detached using a small volume of 0.05% (vol/vol) Trypsin-EDTA solution (Gibco/Thermo Fisher Scientific, Waltham, MA, USA) and a 4 min incubation at 37 °C. Trypsin was deactivated using 5 to 10 mL of complete RPMI 1640 medium and cells were homogenized before being transferred to a conical tube. Intact host cells containing *O. tsutsugamushi* were pelleted via centrifugation for 3 min at 1000× *g*, supernatants were discarded, and infected host cells were resuspended in 3 mL of room temperature PBS. To release intracellular bacteria, 1.5 mL aliquots of infected host cells were mechanically lysed by bead beating with 0.5 mm zirconia beads (BioSpec Products, Bartlesville, OK, USA) for 20 s at 4.0 m/s using a FastPrep-24 bead homogenizer (MP Biomedicals, Irvine, CA, USA). To remove host cell debris, samples were centrifuged for 3 min at 1000× *g* or passed through a 2.0 μm filter attached to a syringe. The supernatant containing intracellular bacteria was transferred to a fresh conical tube for use as inoculum. In infection experiments, host cells were seeded in culture flasks or plates at 90 to 95% confluency and maintained in complete RPMI 1640 medium at 37 °C and 5% CO_2_ for 24 h prior to infection. Host cells were infected with *O. tsutsugamushi* in a small volume of fresh complete RPMI 1640 medium. Bacterial internalization was allowed to proceed with centrifugation (500× *g* for 10–15 min) followed by a 2 h incubation at 37 °C and 5% CO_2_ for plates and flasks, respectively. Following internalization, host cells were washed twice with room temperature PBS to remove unattached and non-internalized bacteria before complete RPMI 1640 medium was added. Infections were incubated at 37 °C in a humidified incubator with 5% CO_2_. For all experiments, infection efficiency (~95%) and infection progression were verified prior to sample processing by immunofluorescence microscopy of infected coverslips immunolabeled with antiserum specific for *O. tsutsugamushi* TSA56 (56 kDa type-specific antigen), which were also used to estimate the multiplicity of infection (MOI) post hoc [26,43].

### 2.3. MTT Assay

EA.hy926 and HeLa cells were seeded in triplicate 96-well plates, one plate per timepoint, at 85 to 90% confluency in complete RPMI 1640 medium. At 24 h post seeding, cells were either left uninfected or incubated with *O. tsutsugamushi* following normal infection protocols. 3-(4,5-dimethylthiazol-2-yl)-2,5-diphenyltetrazolium bromide (MTT) assays were performed on paired uninfected and infected wells as follows. Cells were washed twice with 0.1 mL PBS, after which 0.1 mL of fresh media supplemented with 10 µL of MTT reagent (5 mg mL^−1^ stock) was added. Plates were returned to the incubator for 45 min before 0.1 mL of MTT Solubility Solution (16% [weight/vol] SDS in 40% [vol/vol] dimethylformamide) was added to each well. Samples were thoroughly mixed using a multi-channel pipette before quantification of formazan production by measuring optical density at 570 nm using a SpectraMax iD5 spectrophotometer (Molecular Devices, San Jose, CA, USA). Duplicate 96-well plates were comparably seeded/infected and used as representative samples to assess infection efficiency and progression, and estimate the MOI, by immunofluorescence microscopy as described above. At the indicated timepoints, plates were fixed and permeabilized using ice-cold 100% methanol.

### 2.4. Transmission Electron Microscopy

For visualization of mitochondrial structure, ~2.0 × 10^5^ HeLa or EA.hy926 cells were seeded onto 60 × 15 mm Permanox dishes (Electron Microscopy Sciences, Hatfield, PA, USA). At 24 h post seeding, cells were left uninfected or incubated with *O. tsutsugamushi*. At 48 h post-infection (hpi), samples were washed with PBS and fixed in 2.5% (vol/vol) glutaraldehyde in 0.1 M sodium cacodylate buffer at room temperature followed by dehydration via serial rinses in 30%, 50%, 70%, 80%, and 95% acetone for 5 min each, and three final 10 min rinses in 100% acetone. After dehydration, samples were infiltrated with a 1:1 ratio of 100% acetone and PolyBed 812 resin (Polysciences, Warrington, PA, USA) overnight. Infiltration continued with pure PolyBed 812 resin for 8 h, followed by a second incubation in PolyBed 812 resin overnight. Polymerization occurred at 55–60 °C for two days. Samples were sectioned into 700–900 Å thick slices using a Leica EM UC7 Ultramicrotome (Leica Microsystems, Durham, NC, USA). Sections were placed on copper grids and stained with 5% uranyl acetate and Reynold’s lead citrate. Grids were visualized using a JEOL JEM-14000Plus transmission electron microscope (JEOL, Tokyo, Japan) with the Gatan OneView 4K × 4K direct electron CMOS camera (Gatan, Pleasanton, CA, USA).

### 2.5. DNA Extraction and Quantification of Mitochondrial DNA Content

Total DNA was extracted from duplicate wells of a 6-well plate containing uninfected or *O. tsutsugamushi*-infected HeLa and EA.hy926 cells. Briefly, coverslips from each sample well were transferred to clean wells and processed by immunofluorescence microscopy to assess infection efficiency and progression, and estimate the MOI, as described above. Spent media from sample wells was discarded and cell monolayers washed twice with 1 mL room temperature PBS. Monolayers were then harvested via trypsinization, transferred to a microcentrifuge tube, and pelleted via centrifugation for 5 min at 1000× *g*, 4 °C. Samples were stored at −20 °C and MOI determined before DNA was extracted using a NucleoSpin Tissue kit (Macherey-Nagel, Bethlehem, PA, USA). DNA was extracted from all uninfected samples. DNA was extracted from infected samples only if at least 90% of the population was infected with roughly an MOI of 10–20 at 24 hpi. DNA was isolated, washed, and eluted in 75 μL of 70 °C molecular grade water. DNA concentration was determined, samples normalized to 20 ng μL^−1^, and 2 μL of each sample added to triplicate wells of a 384-well qPCR plate. Mitochondrial DNA content was then measured by calculating the ratio of mitochondrial:nuclear gene copy number as determined via qPCR using a CFX-384 real-time PCR detection system (Bio-Rad, Hercules, CA, USA) and the iTaq Universal SYBR green Supermix (Bio-Rad, Hercules, CA, USA). For these efforts, 100 base pair amplicons were detected using primers designed to the human nuclear genes hemoglobin subunit beta (HHB) (5′-CTGCTGGTGGTCTACCCTTG-3′ and 5′-AGCGTCCCATAGACTCACCC-3′) and β2-microglobulin (β2M) (5′-TGTAAGCAGCATCATGGAGGT-3′ and 5′-CATACCTGGGGCCATACACC-3′), in addition to the mitochondrial encoded genes cytochrome C oxidase 1 (CO1) (5′-CCAATACCAAACGCCCCTCT-3′ and 5′-AGAATGGGGTCTCCTCCTCC-3′) and NADH dehydrogenase 1 (ND-1) (5′-TATGACGCACTCTCCCCTGA-3′ and 5′-GTAGCGGAATCGGGGGTATG-3′).

### 2.6. Generation of Cytosolic and Mitochondrial Protein Fractions

Separation of cytosolic and mitochondrial protein pools were accomplished using the ProteoExtract Subcellular Proteome Extraction Kit (Millipore Sigma, Burlington, MA, USA) per manufacturer’s instructions for frozen cell pellets. Uninfected and *O. tsutsugamushi*-infected monolayers were harvested at indicated timepoints via trypsinization and pelleted by centrifugation at 1000× *g* for 3 min, 4 °C. Pellets were washed twice with ice-cold Wash Buffer (Millipore Sigma, Burlington, MA, USA) and pellets were stored at −20 °C until infection characterization. Pellets from usable samples were thawed at room temperature before being resuspended in 0.5 mL ice-cold Extraction Buffer I containing 2.5 μL Protease Inhibitory Cocktail (Millipore Sigma, Burlington, MA, USA). Samples were incubated for 10 min at 4 °C on a rotating mixer. Insoluble materials were pelleted via centrifugation at 500× *g* for 10 min, 4 °C. Supernatants containing cytosolic proteins were transferred to fresh tubes while the pellet was resuspended in 0.5 mL ice-cold Extraction Buffer II containing 2.5 μL Protease Inhibitory Cocktail (Millipore Sigma, Burlington, MA, USA). Samples were mixed well and incubated for 10 min at 4 °C on a rotating mixer. Insoluble materials were pelleted via centrifugation at 6000× *g* for 10 min, 4 °C. Supernatants containing mitochondrial proteins were transferred to fresh tubes. The remaining pellets containing nuclear and cytoskeletal proteins were lysed using a modified Radioimmunoprecipitation assay (RIPA) buffer (50 mM Tris-HCl pH 7.4, 150 mM NaCl, 1 mM EDTA, 1.0% (vol/vol) NP-40, 0.25% (vol/vol) sodium deoxycholate) in an ice bath for 1 h with brief vortexing every 10 min. Insoluble proteins were removed by centrifugation at 16,000× *g* for 10 min at 4 °C. All fractions were stored at −20 °C until use.

### 2.7. Western Blot Analysis

To process samples for SDS-PAGE, normalized protein lysates were mixed with 1X SDS-PAGE loading buffer (42 mM Tris-HCl pH 6.8, 9% (vol/vol) glycerol, 34.6 mM SDS, 38 μM bromophenol blue, 350 mM 2-mercaptoethanol) and heated for 10 min at 100 °C. Proteins were separated by SDS-PAGE in 4–15% mini-Protean gels (Bio-Rad, Hercules, CA, USA) at 100 V for 20 min, followed by 200 V for 30–45 min. Proteins were transferred onto nitrocellulose membranes in ice-cold Towbin buffer at 100 V for 30 min. For immunoblotting, membranes were washed in Tris-buffered saline containing 0.05% (vol/vol) Tween-20 (TBS/T) and blocked in TBS/T containing 5% (weight/vol) nonfat dry milk. Membranes were incubated overnight at 4 °C with the following primary antisera and antibodies: TSA56 rabbit antiserum (1:3000) [44], GAPDH monoclonal antibody (1:4000 [sc-365062; Santa Cruz, Santa Cruz, CA, USA]), β-actin monoclonal antibody (1:4000 [sc-47778; Santa Cruz, Santa Cruz, CA, USA]), pan-VDAC monoclonal antibody (1:2000 [sc-390996; Santa Cruz, Santa Cruz, CA, USA]), VDAC1 monoclonal antibody (1:1000 [MA5-33205; Invitrogen, Carlsbad, CA, USA]), VDAC1/VDAC3 monoclonal antibody (1:1000 [ab14734; abcam, Waltham, MA, USA]), VDAC2 polyclonal antibody (1:2000 [PA5-28106; Invitrogen, Carlsbad, CA, USA]), VDAC3 polyclonal antibody (1:1000 [PA5-100190; Invitrogen, Carlsbad, CA, USA]), PRDX3 monoclonal antibody (1:2000 [MA5-32721; Invitrogen, Carlsbad, CA, USA]), and TOM20 monoclonal antibody (1:1000 [sc-17764; Santa Cruz, Santa Cruz, CA, USA]). Secondary antibodies were horseradish peroxidase-conjugated to anti-mouse IgG or anti-rabbit IgG (1:10,000 [Cell Signaling Technology, Danvers, MA, USA]) and were incubated with blots for 1 h at room temperature. All blots were incubated with SuperSignal West Pico PLUS, SuperSignal West Dura PLUS, or SuperSignal West Femto PLUS chemiluminescent substrates (ThermoFisher Scientific, Waltham, MA, USA) as per manufacturer’s instructions. Blots were imaged using ChemiDoc Touch Imaging System (Bio-Rad, Hercules, CA, USA) and processed with Bio-Rad Image Lab 6.0 software.

### 2.8. Rat TSA56 Antiserum Generation

A histidine-tagged chimeric antigen consisting of the *O. tsutsugamushi* str. Ikeda TSA56 hydrophilic segments 105 to 203, 243 to 354, and 360 to 420 was generated exactly as described [44]. In total, 50 μg of this antigen emulsified in a 1:1 ratio with complete Freund adjuvant in a total volume of 400 μL was used to immunize an 8-week-old female Sprague-Dawley rat (Charles River, Wilmington, MA, USA). At weeks 3 and 5, the rat was boosted with 25 μg of protein in incomplete Freund adjuvant. At week 6, the rat was euthanized by CO_2_ asphyxiation, blood was collected by cardiac puncture, and the TSA56 antiserum was recovered. Rats were housed no less than two per cage to provide social interaction. Control antiserum was collected prior to immunization. The experimental point was determined by measuring antibody titers in immunized animals. All animals were monitored daily for pain or distress, including dehydration, posture, swelling, prolapse, weight loss, injection site edema, labored breathing, alopecia, locomotion defects, lethargy, abnormal discharge, head tilt, respiration issues, and other abnormalities. The moribundity scale was used as a guideline to inform decisions regarding humane endpoint. If a rat exhibited a change in one or more of the above criteria that scores at >1 on the moribundity scale, then the humane endpoint would have been reached and the rat euthanized.

### 2.9. Immunofluorescence Microscopy

HeLa and EA.hy926 cells were seeded on 12 mm glass coverslips (Electron Microscopy Sciences, Hatfield, PA, USA) in 24-well plates and infected with *O. tsutsugamushi*. At specified timepoints, coverslips were washed twice with PBS before cells were fixed using 100% ice-cold methanol (Fisher Scientific, Pittsburgh, PA, USA) for 10 min at −20 °C or 2% (vol/vol) paraformaldehyde (PFA; Electron Microscopy Sciences, Hatfield, PA, USA) in PBS overnight at 4 °C. PFA-fixed samples were permeabilized using either 0.1% (vol/vol) Triton X-100 (Fisher Scientific, Pittsburgh, PA, USA) in PBS or 100% ice-cold methanol (Fisher Scientific, Pittsburgh, PA, USA) for 10 min prior or to immunolabeling. For immunolabeling, samples were rinsed three times in PBS, followed by incubation in primary antibody for 1 h in 1% (weight/vol) bovine serum albumin (BSA) in PBS. Primary antisera and antibodies used in this study were: rabbit anti-TSA56 [44], rat anti-TSA56 (1:2000), anti-pan-VDAC (1:1000 [sc-390996; Santa Cruz, Santa Cruz, CA, USA]), anti-VDAC1/VDAC3 (1:1000 [ab14734; abcam, Waltham, MA, USA]), anti-PRDX3 (1:2000 [MA5-32721; Invitrogen, Carlsbad, CA, USA]), and anti-TOM20 (1:1000 [sc-17764; Santa Cruz, Santa Cruz, CA, USA]). Following incubation with primary, samples were rinsed three times with PBS and subsequently incubated for 1 h with Alexa Fluor 488- or 594-conjugated chicken or goat anti-rabbit IgG, Alexa Fluor 488- or 594-conjugated chicken or goat anti-rat IgG, Alexa Fluor 488- or 594-conjugated chicken or goat anti-mouse IgG, Alexa Fluor 633-conjugated goat anti-rabbit IgG, Alexa Fluor 633-conjugated goat anti-mouse IgG, or Alexa Fluor 700-conjugated goat anti-rabbit IgG (ThermoFisher Scientific, Waltham, MA, USA) at a 1:1000 dilution in a small volume of 1% (weight/vol) bovine serum albumin (BSA) in PBS containing 0.1 µg mL^−1^ 4′,6-diamidino-2-phenylindole (DAPI; ThermoFisher Scientific, Waltham, MA, USA). Three final rinses were performed, and coverslips were mounted with Prolong Gold or Prolong Diamond Anti-Fade reagent (ThermoFisher Scientific, Waltham, MA, USA).

Fluorescent micrographs were acquired using a Leica DMi8 inverted epifluorescence microscope (Leica Microsystems, Durham, NC, USA) equipped with an Andor iXon Life 888 electron-multiplying charge-coupled device (EMCCD) camera (Oxford Instruments, Oxfordshire, UK) and EL6000. A 100×/1.3 NA oil immersion objective lens was used. Additionally, confocal micrographs were acquired with a Leica DMi8 inverted laser-scanning confocal microscope (Leica Microsystems, Durham, NC, USA) equipped with a Stellaris8/Falcon system and white-light laser. A 63×/1.4 NA oil immersion objective lens was used. SIM micrographs were acquired with a ZEISS Elyra 7 SIM/STORM (ZEISS Microscopy, Jena, Germany) equipped with two PCO edge sCMOS cameras. A 63×/1.4 NA oil immersion objective lens was used. Image processing was performed using the Leica LAS X software (v3.74.23463; Leica Microsystems, Durham, NC, USA), ZEISS ZEN Blue software (v3.0; ZEISS Microscopy, Jena, Germany), and Fiji [45] (v2.16.0/1.54p).

### 2.10. Sequence Homology Analyses of VDAC Paralogs and VDAC Antibody Immunogens

In silico sequence homology analyses were performed using the NCBI Basic Local Alignment Search Tool (BLAST v2.17.0). Human VDAC1 (NM_003374.3; NP_003365.1), VDAC2 (NM_001391963.1; NP_001378892.1), and VDAC3 (NM_005662.7; NP_005653.3) nucleotide and amino acid sequences were queried against the *O. tsutsugamushi* Ikeda genome and proteome (NC_010793.1; taxid: 334380). BLASTn/BLASTp analyses were conducted using default parameters. To evaluate potential antibody cross-reactivity, full-length and 10–17–amino acid regions for the immunogen sequences used to generate the pan-VDAC (sc-390996, Santa Cruz) and VDAC2 (aa 229–294; PA5-28106, Invitrogen) antibodies were analyzed against the *Orientia* proteome using BLASTp. In cases where limited similarity was detected, aligned regions were further examined for identity length.

### 2.11. Statistical Analysis and Data Presentation

Statistical analyses were performed using GraphPad Prism (v10.6.1; Dotmatics, Boston, MA, USA). Independent *t*-tests were performed and *p*-values reported for those experiments comparing only two groups. For experiments comparing more than two groups, one-way analysis of variance (ANOVA), followed by a Dunnett’s multiple comparison test, was performed. Alpha was set to 0.05 for all statistical analyses, and data normality was assessed using the Shapiro–Wilk test. Figures were assembled in Amadine (v1.7; Belight Software, Inc., Chicago, IL, USA) and PowerPoint (v16.103; Microsoft, Redmond, WA, USA).

## 3. Results

### 3.1. Mitochondria Are Enzymatically and Structurally Aberrant in O. tsutsugamushi-Infected Cells

**Figure 1:**
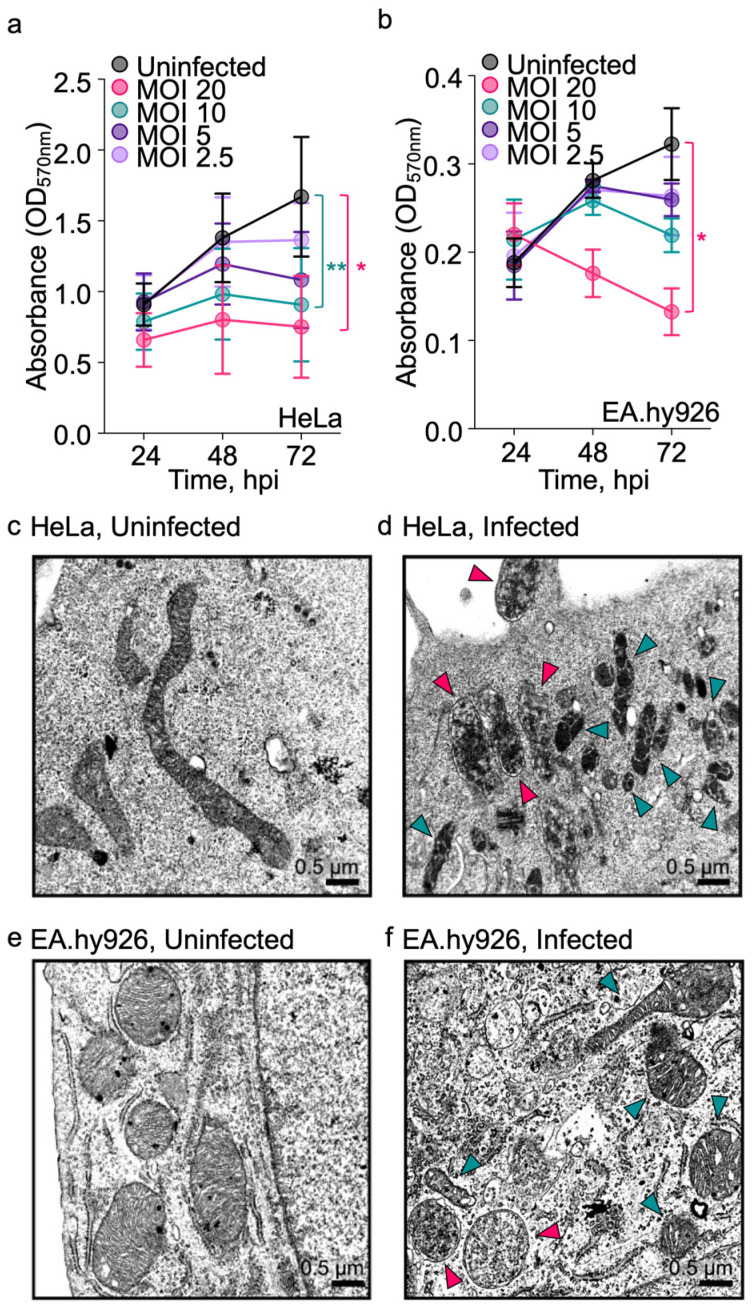
Mitochondria exhibit impaired enzymatic activity and structural abnormalities during *O. tsutsugamushi* infection. (**a**) HeLa and (**b**) EA.hy926 cells were left uninfected or infected with *O. tsutsugamushi* at multiplicities of infection (MOIs) of 2.5, 5, 10, or 20. At the indicated hours post-infection (hpi), mitochondrial enzymatic activity was quantified by MTT assay. Data illustrate means ± SEM (*N* = 3–4). Statistical significance was determined by one-way ANOVA with Dunnett’s post hoc test; *, *p* < 0.05; **, *p* < 0.002. Transmission electron micrographs of uninfected and infected (**c**,**d**) HeLa and (**e**,**f**) EA.hy926 cells at 48 hpi. Pink and teal arrows indicate *O. tsutsugamushi* organisms and mitochondria, respectively. Scale bars, 1 μm. *N* = 2.

*O. tsutsugamushi*-infected endothelial cells exhibit reduced concentrations of mitochondrial-derived and mitochondrial-regulated metabolites, including amino acids, TCA intermediates, and lipids [26]. These data, compounded with the significant decline in mitochondrial redox potential that occurs by 24 h post-infection (hpi) [26], suggest that *O. tsutsugamushi* impairs mitochondrial function. We assessed if mitochondrial integrity was bacterial burden-dependent using a MTT assay and two proven infection models, cervical epithelial-like HeLa and hybridoma endothelial-like EA.hy926 cells [26,29,46,47,48,49,50]. Mitochondrial enzymatic activity measured by formazan production was significantly reduced at 24 hpi in HeLa cells infected at a multiplicity of infection (MOI) of 10 or 20 and continued to decline throughout the experiment (Figure 1a). At MOIs of 2.5 or 5, mitochondrial activity only trended to decline at 48–72 hpi. Similar results were observed for EA.hy926 cells except that only the MOI of 20 condition resulted in a significant reduction in formazan production at 72 hpi (Figure 1b). Of note, the MTT assay measures cellular reductase activity, which is largely driven by mitochondrial dehydrogenases but can reflect contributions from other cellular oxidoreductases. Thus, the observed differences in basal formazan production between cell lines likely reflect inherent variations in the metabolic activity and mitochondrial content of each cell type.

The observed decline in mitochondrial enzymatic activity prompted us to assess mitochondrial morphology using transmission electron microscopy (TEM). Mitochondria in uninfected HeLa cells exhibited tubular morphologies and defined, regularly spaced cristae (Figure 1c). By comparison, at 48 hpi HeLa cells that had been infected with *O. tsutsugamushi* at an MOI of 15–20 contained mitochondria that were fragmented or misshaped and had irregular cristae, indicating organelle stress [51] (Figure 1d). Results were comparable for EA.hy926 cells, where uninfected cells had mitochondria that were largely spheroid and infected cells had mitochondria exhibiting irregular shape and cristae (Figure 1e,f). In contrast to earlier reports, infected cells did not appear heavily vacuolated or devoid of cytoskeletal structures [15], which may reflect our use of synchronized infections and a lower MOI than in those studies. Thus, *O. tsutsugamushi* induces mitochondrial enzymatic and structural aberrancy in a dose-dependent manner.

### 3.2. O. tsutsugamushi Does Not Alter Mitochondrial Abundance or the Levels of Critical Mitochondrial Proteins

**Figure 2:**
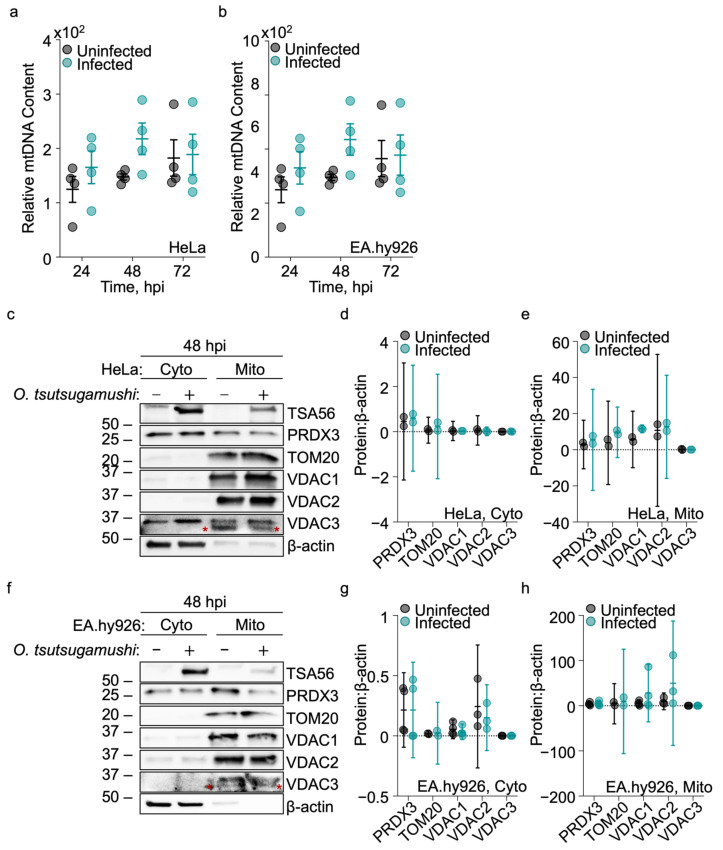
Mitochondrial DNA and homeostatic protein content remain unchanged during *O. tsutsugamushi* infection. (**a**) HeLa and (**b**) EA.hy926 cells were left uninfected or infected with *O. tsutsugamushi*. At 24, 48, and 72 hpi, total DNA was extracted and the ratio of mitochondrial (mtDNA) to nuclear (nucDNA) genes determined by qPCR. Bars represent mean ± SEM. Symbols depict independent experiments (*N* = 3–4). Immunoblots of cytosolic (Cyto) and mitochondrial (Mito) fractions from uninfected and infected (**c**) HeLa and (**f**) EA.hy926 cells at 48 hpi. Blots were immunolabeled for mitochondrial PRDX3, TOM20, VDAC1, VDAC2, and VDAC3. *O. tsutsugamushi* TSA56 served as confirmation of infection. β-actin was a loading control. Red asterisks (*) denote VDAC3 in Mito fractions. Mean normalized ratios ± 95% CI of PRDX3, TOM20, VDAC1, VDAC2, and VDAC3 to β-actin were determined by densitometry for Cyto and Mito fraction of uninfected and infected (**d**,**e**) HeLa and (**g**,**h**) EA.hy926. *N* = 2–4.

Because our data indicated mitochondrial impairment during *O. tsutsugamushi* infection, additional aspects of mitochondrial health and function were examined. We first quantified the ratio of mitochondrial DNA (mtDNA) to nuclear DNA (nucDNA), which correlates to the number of mitochondria per cell [52,53,54]. DNA extracted from uninfected and *O. tsutsugamushi*-infected HeLa and EA.hy926 cells at 24, 48, and 72 hpi was subjected to qPCR analysis. The relative mtDNA content was comparable between uninfected and infected cells at each timepoint (Figure 2a,b), suggesting no difference in mitochondrial numbers.

We next assessed the relative content of mitochondrial proteins critical for organelle homeostasis. These were the mitochondria-specific peroxiredoxin (PRDX3), an antioxidant that is essential for mitochondrial function and quality control [55]; the receptor subunit of the translocase of outer mitochondrial membrane (TOM) complex, TOM20, which is essential for mitochondria protein import and whose expression can change in certain diseases [56,57]; and VDAC, which controls the transport of ions and metabolites, in addition to serving as an interacting partner with other organelle proteins to regulate broad cellular functions [35,58]. Because each protein is synthesized in the cytosol before mitochondrial import, cytosolic and mitochondrial fractions from uninfected or *O. tsutsugamushi*-infected host cells were isolated and relative protein content determined via immunoblot. Additionally, we probed for VDAC paralogs, VDAC1, VDAC2, and VDAC3, which are evolutionary divergent and exhibit distinct functions and tissue tropisms [59,60]. Antisera against the abundant *O. tsutsugamushi* outer membrane protein TSA56 (56 kDa type-specific antigen) was used to confirm *O. tsutsugamushi* infection [44]. TOM20, VDAC1, VDAC2, and VDAC3 were present in the mitochondrial but not cytosolic fractions, thus confirming fraction purity. PRDX3 was detectable in the cytosolic fractions for both uninfected and infected HeLa and EA.hy926 cells, immortalized lines that exhibit higher baseline levels of reactive oxygen species [61,62]. Although PRDX3 is canonically localized to the mitochondrial matrix, previous reports indicate that oxidative stress can promote PRDX3 redistribution or release into the cytosol [55,63], which may account for its detection in these fractions. At 48 hpi—a timepoint corresponding with robust bacterial growth and moderate reduction in mitochondrial redox potential—PRDX3, TOM20, and VDAC paralog levels were comparable between uninfected and infected cells (Figure 2c–h). Because steady-state protein levels of PRDX3, TOM20, and the VDAC paralogs were unchanged during infection, corresponding transcriptional differences were not anticipated [64,65]. Overall, while mitochondria become enzymatically and structurally impaired during *O. tsutsugamushi* proliferation, neither mitochondrial number nor the abundance of homeostatic proteins are reduced.

### 3.3. O. tsutsugamushi Selectively Associates with VDAC1 and VDAC3 Immunosignal

**Figure 3:**
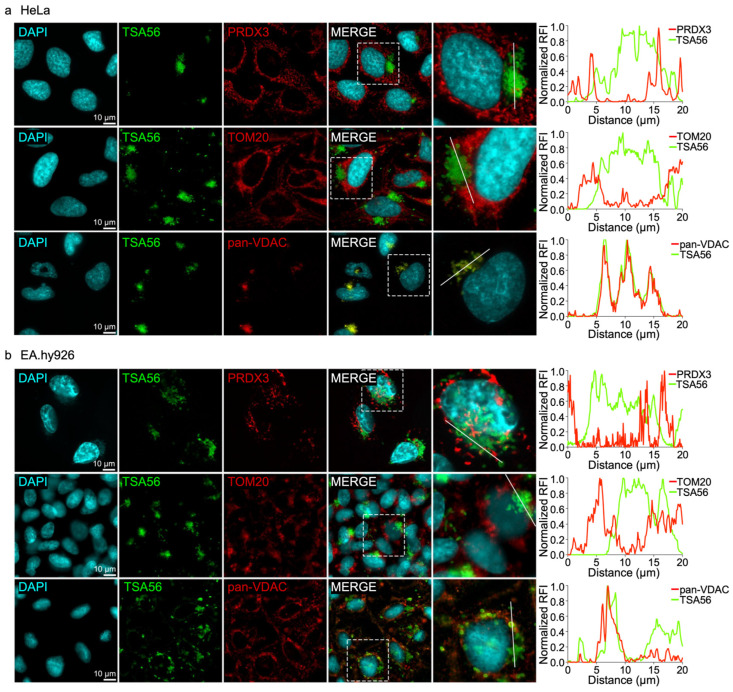
Immunosignals for mitochondrial VDAC but not PRDX3 or TOM20 localize with *O. tsutsugamushi*. Immunofluorescence micrographs of (**a**) HeLa and (**b**) EA.hy926 cells infected with *O. tsutsugamushi*. At 48 hpi, samples were fixed, stained with DAPI (host cell nuclei and bacterial nucleoids; cyan), and immunolabeled for *O. tsutsugamushi* TSA56 (green) and mitochondrial PRDX3, TOM20, or pan-VDAC (red). Visualization was performed using epifluorescence microscopy. Regions boxed by hatch lines in merged micrographs are magnified in the final micrograph of each series. Paired line scan intensity profiles were generated across regions of interest intersecting *O. tsutsugamushi* and adjacent host mitochondria. Fluorescence intensities for each channel (TSA56 and the indicated mitochondrial protein) were normalized to their respective maximum values and plotted as relative fluorescence intensity (RFI). Scale bars, 10 µm. *N* = 5.

**Figure 4:**
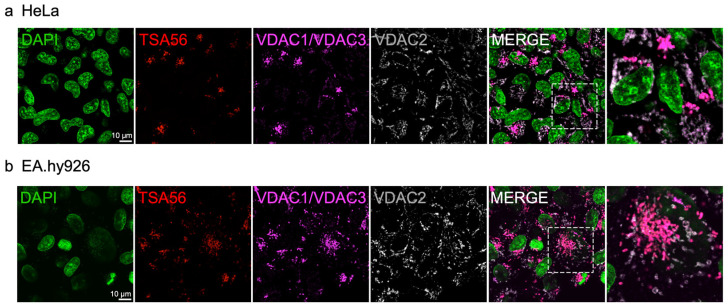
VDAC1 and VDAC3, but not VDAC2 colocalize with *O. tsutsugamushi*. Confocal micrographs of *O. tsutsugamushi*-infected (**a**) HeLa and (**b**) EA.hy926 cells. At 48 hpi, samples were fixed, stained with DAPI (host cell nuclei and bacterial nucleoids; green), and immunolabeled for TSA56 (red), VDAC1/VDAC3 (magenta), and VDAC2 (gray). Regions boxed by hatch lines in the merged micrographs are magnified in the final micrograph of the series. Scale bars, 10 µm. *N* = 3.

**Figure 5:**
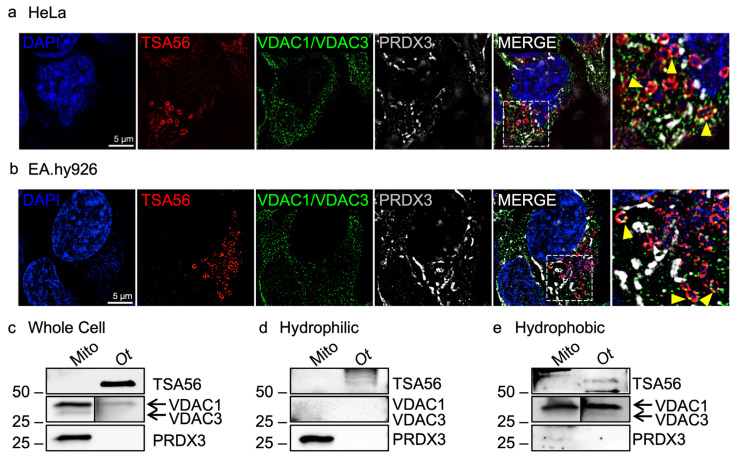
VDAC1 and VDAC3 immunosignals associate with the *O. tsutsugamushi* membrane. (**a**) HeLa or (**b**) EA.hy926 cells were infected with *O. tsutsugamushi*. At 48 hpi, samples were fixed, stained with DAPI (host cell nuclei and bacterial nucleoids; blue), and immunolabeled for *O. tsutsugamushi* TSA56 (red), VDAC1/VDAC3 (green), PRDX3 (gray), and analyzed via structured illumination microscopy. Regions boxed by hatch lines in the merged micrographs are magnified in the final micrograph of each series. Yellow arrows indicate punctate VDAC1/VDAC3 immunolabel colocalizing with that for TSA56. Scale bars, 5 µm. *N* = 3. Immunoblots from (**c**) whole cell, (**d**) hydrophilic, and (**e**) hydrophobic protein fractions from isolated HeLa mitochondria or host cell-free *O. tsutsugamushi* were probed with PRDX3, VDAC1/VDAC3, and TSA56 antibodies. Arrows denote VDAC1 versus VDAC3 in samples. *N* = 5.

Next, we visualized the spatial localization of PRDX3, TOM20, and VDAC in infected cells. PRDX3 and TOM20 antibodies distinctly immunolabeled mitochondria throughout the cytosol, which surrounded perinuclear *O. tsutsugamushi* microcolonies in both HeLa and EA.hy926 cells at 48 hpi (Figure 3). Intriguingly, antibody targeting a conserved region across VDAC paralogs 1–3 (pan-VDAC) predominantly detected *O. tsutsugamushi* in infected HeLa cells and immunolabeled both mitochondria and *Orientia* organisms in infected EA.hy926 cells. To further investigate this phenomenon, we assessed whether immunosignal colocalization between VDAC and TSA56 was paralog specific. Because the VDAC3 antibody employed for the immunoblot analyses above proved to be incompatible with immunofluorescence microscopy, we utilized a VDAC1/VDAC3 monoclonal antibody commercially validated to detect each paralog. Infected HeLa and EA.hy926 cells probed for VDAC1/VDAC3, VDAC2, and TSA56 were examined by confocal microscopy. Whereas VDAC1/VDAC3 and VDAC2 antibodies immunolabeled mitochondria, only anti-VDAC1/VDAC3 detected *O. tsutsugamushi* (Figure 4). VDAC2 immunosignal colocalization with *Orientia* was infrequently observed and only when the bacteria were immediately adjacent to mitochondria, suggesting proximity-based signal convergence rather than specific colocalization. Employing structured illumination microscopy (SIM) further confirmed VDAC1/VDAC3 colocalization. As expected, PRDX3 and VDAC1/VDAC3 antibodies immunolabeled mitochondria (Figure 5a,b). VDAC1/VDAC3 immunosignal was also present and interspersed along the periphery of *O. tsutsugamushi* organisms, frequently colocalizing with TSA56. Thus, immunosignal for VDAC1/VDAC3 but not VDAC2, TOM20, or PRDX3 colocalizes with the membrane of cytosolic *O. tsutsugamushi* bacteria.

### 3.4. VDAC1 and VDAC3 Are Detected in O. tsutsugamushi Membrane Fractions

As a complimentary approach, *O. tsutsugamushi* organisms that had naturally egressed from HeLa cells were recovered and resolved into hydrophilic and hydrophobic fractions, the latter of which was enriched for inner and outer membranes. Mitochondria isolated from uninfected cells were processed in parallel as a control. Whole cell, hydrophilic, and hydrophobic fractions were subjected to immunoblot analysis using VDAC1/VDAC3, PRDX3, and TSA56 antibodies. As expected, TSA56 was in the *O. tsutsugamushi* whole cell lysate (WCL), enriched in the hydrophobic fraction versus the hydrophilic fraction, and absent from all fractions derived from mitochondria (Figure 5c–e). PRDX3, a mitochondrial matrix protein, was present in the mitochondrial WCL and heavily enriched in the hydrophilic fraction compared to the hydrophobic fraction. PRDX3 was absent in all *O. tsutsugamushi* samples. These results confirmed both mitochondrial and *O. tsutsugamushi* fraction purity. Despite being the same size, VDAC3 exhibits a faster electrophoretic mobility than VDAC1 due to differences in cysteine content [60,66,67]. VDAC1 has an observed molecular weight of ~30–35 kDa while that for VDAC3 is ~28–31 kDa. The VDAC1/VDAC3 antibody detected proteins of expected molecular weights for each paralog in the mitochondrial WCL and hydrophobic fractions. The greater abundance of the VDAC1 band is consistent with what has been reported for mammalian cells [59,60,68]. Notably, VDAC1 and VDAC3 were detected in *Orientia* WCL and hydrophobic fractions at relative ratios comparable to those observed for mitochondria. Overall, VDAC1 and VDAC3 appear to associate with *O. tsutsugamushi* membranes.

### 3.5. Assessment of Potential VDAC Antibody Cross-Reactivity in O. tsutsugamushi

Based on genome annotations, no *O. tsutsugamushi* strain is predicted to encode a VDAC homolog [64,65,66,67]. The pan-VDAC antibody (Santa Cruz sc-390996) that recognized *Orientia* via immunofluorescence microscopy (Figure 3) was raised against a 40-residue immunogen that is conserved between VDAC1, VDAC2, and VDAC3 (Table 1). The VDAC1/VDAC3 antibody (abcam ab14734) that detected *Orientia* via immunofluorescence microscopy and proteins of the expected size for VDAC1 and VDAC3 in bacterial membrane fractions (Figure 4 and Figure 5) is stated by the vendor to have been raised against full-length VDAC1 and VDAC3. We considered the possibility that the observed immunolabeling by these antibodies could be due to their cross-reactivity with *Orientia* membrane proteins. Accordingly, we used the NCBI Basic Local Alignment Search Tool (BLAST) to query full-length VDAC1, VDAC2, and VDAC3 against the *O. tsutsugamushi* Ikeda proteome (NC_010793.1; taxid: 334380). No significant sequence similarities were identified (Table 1). Querying the pan-VDAC immunogen also yielded no hits. When four successive 10-amino acid segments of this sequence were queried, each exhibited homology with four- to six-residue stretches of several *O. tsutsugamushi* proteins predicted to be membrane- and non-membrane-localized. None of these proteins share the same theoretical size as VDAC1 or VDAC3.

As a negative control, we also performed searches using the 66-residue immunogen for the VDAC2 antibody (Invitrogen PA5-28106) that does not recognize *O. tsutsugamushi* (Figure 4) and truncated segments thereof. Like that observed for the pan-VDAC immunogen, only short peptides of the VDAC2 immunogen but not the full-length sequence yielded hits of significant similarity with *O. tsutsugamushi* membrane and non-membrane proteins. While not absolute, these results together with the expected size for VDAC1 and VDAC3 in the *O. tsutsugamushi* hydrophobic fractions argue that *O. tsutsugamushi* recognition by the VDAC1 and VDAC1/VDAC3 antibodies are due to selective association of these host proteins with the bacterium and not cross-reactivity.

## 4. Discussion

Our findings reveal that *O. tsutsugamushi* diminishes mitochondrial enzymatic activity and promotes irregular cristae organization, despite stable abundance of key mitochondrial proteins, including PRDX3, TOM20, and VDAC paralogs 1, 2, and 3. Hence, mitochondrial function is perturbed during infection without overt loss of organelle content. The bacterial dose-dependent impairment of mitochondrial enzymatic activity is consistent with a close metabolic and spatial relationship between *O. tsutsugamushi* and mitochondria that may reflect competition for shared metabolic resources such as amino acids, TCA intermediates, and ATP rather than organelle destruction. Importantly, immunofluorescence microscopy and immunoblot analysis of *O. tsutsugamushi* whole cell and membrane fractions demonstrated a selective spatial *Orientia*-VDAC association. While this association requires further validation, these observations implicate VDAC as a potential node at the host mitochondrial–*O. tsutsugamushi* interface.

Mitochondrial functions are frequently modulated by intracellular pathogens given the critical role this organelle plays in eukaryotic cellular metabolism, homeostasis, and innate immune signaling [34,69]. While viruses tend to indirectly manipulate mitochondrial regulatory pathways, bacterial pathogens often directly induce mitochondrial dysfunction [70,71,72]. In this context, the endosymbiotic origin of mitochondria from an alphaproteobacterial ancestor provides an evolutionary framework for why bacterial proteins specifically interface with mitochondria. Several bacterial pathogens modulate mitochondrial dynamics, including fission and fusion, which ultimately alters mitochondrial homeostasis. *Legionella pneumophila* and *Salmonella enterica* serovar Typhimurium deploy effectors to induce mitochondrial fragmentation, a process that shifts host cellular metabolism [73,74,75]. Bacterial toxins target mitochondrial inner membrane components or electron transport chain complexes leading to increased mitochondrial calcium levels, organelle depolarization, and cytochrome c release, all of which are hallmark triggers of apoptosis or necrosis [70,72,76,77,78]. These strategies differ from what we observed during *O. tsutsugamushi* infection, where mitochondrial morphology and content were largely preserved despite irregular cristae organization. Combined with evidence that *O. tsutsugamushi* infection impairs cellular ATP production [26] and intracellular calcium release [38], this suggests that *O. tsutsugamushi* infection progressively elicits mitochondrial metabolic stress.

As an obligate intracellular bacterium with extensively reduced metabolic capacity, *O. tsutsugamushi* is thought to rely heavily on host-derived metabolites to support replication. Central carbon metabolism intermediates and amino acids are considered the most relevant host-derived nutrients for *O. tsutsugamushi* growth, as inferred from genome-based metabolic reconstructions and predicted transport systems [79,80,81,82]. Indeed, *O. tsutsugamushi* intracellular replication coincides with perturbations in Golgi-to-endoplasmic reticulum (ER) trafficking and ER-associated degradation (ERAD), processes that increase protein turnover to yield free amino acids [44,83]. Notably, the ER and mitochondria are physically linked via mitochondria-associated membranes (MAMs), which supports the transfer of lipids, calcium, and signaling molecules between the two organelles [84]. MAMs connect cellular functions of these two organelles, where stress or impaired integrity in one directly activates a response in the other [85,86]. Because mitochondria serve as central hubs for amino acid metabolism, lipid biosynthesis, and redox balance (e.g., ATP production), sustained diversion or depletion of these metabolic pools via competition could constrain mitochondrial activity, thereby influencing ER processes. Consistent with this model, several host central carbon metabolites including amino acids and TCA cycle intermediates are progressively reduced during *O. tsutsugamushi* infection [26].

Passive competition, whereby one organism restricts nutrient availability to the detriment of another, is a well-described phenomenon [87,88,89]. *Escherichia coli* can shift its metabolic program to enhance nutrient uptake and thereby limit resource availability for competitors [90,91]. Other bacteria secrete secondary metabolites that sequester or degrade nutrients, such as siderophores (e.g., *Pseudomonas aeruginosa*) or digestive enzymes (e.g., *Bacteroides* spp.) [92,93,94,95,96,97]. Although these examples are drawn from microbial communities, they illustrate general principles of resource-based competition that analogously operate within the mammalian cytosol. Indeed, intracellular bacteria including *Chlamydia trachomatis* and *Shigella flexneri* recruit mitochondria or localize near mitochondrial networks during replication [98]. In these systems, mitochondrial proximity has been proposed to facilitate access to host-derived metabolites, modulate redox balance, or influence innate immune signaling, often without invoking effector-mediated organelle damage or loss.

The selective spatial association observed between *O. tsutsugamushi* and the mitochondrial channel proteins, VDAC1 and VDAC3, is notable in the context of metabolic interference rather than destruction. Given the role of VDAC in regulating nutrient and ion flux, as well as organelle communication [58,60,99], this spatial association may reflect regions of heightened metabolic exchange or signaling at the host–pathogen interface. Such an arrangement would be compatible with the prolonged intracellular replication of *O. tsutsugamushi* and its reported ability to delay host cell death [29,36,37,38,39,40,41]. Previous studies have alluded to *Rickettsia prowazekii* and *R. canadensis* incorporating VDAC into bacterial membranes [100,101], a hypothesis that has been widely cited given the close evolutionary relationship between *Rickettsiae* and mitochondria, overlapping metabolic requirements, and limited predicted transport systems [81]. Mitochondrial VDAC proteins are β-barrel OMPs that, despite lacking primary sequence homology with proteobacterial porins, share striking similarities in overall structure and assembly. Moreover, recombinant mitochondrial VDAC has been shown to assemble in bacterial outer membranes [102] as well as the reciprocal experiment [103,104,105,106], indicating that core β-barrel assembly pathways are evolutionarily conserved. Whether *Rickettsia* and *Orientia* spp. naturally recognize and incorporate host-derived OMPs during infection remains unclear.

The approaches used here do not resolve whether the observed VDAC1/VDAC3-TSA56 immunosignal colocalization reflects direct molecular interaction, transient membrane engagement, or close spatial juxtaposition at the host–pathogen interface. Although SIM provides enhanced spatial resolution, it cannot discriminate membrane incorporation from closely apposed structures. However, the complementary immunoblot analyses argue further for VDAC1/VDAC3 association with the *O. tsutsugamushi* membrane. Indeed, the anti-VDAC1/VDAC3 immunoreactive bands were present in the western-blotted *O. tsutsugamushi* membrane fraction and of the same size and relative abundances as the immunoreactive bands in the mitochondrial membrane fraction. While we cannot absolutely rule out that the SIM and Western blot results are not due to VDAC1/VDAC3 antibody cross-reactivity with *O. tsutsugamushi* proteins, none of *Orientia* hits that exhibited limited sequence similarity at the level of short linear epitopes were of the same theoretical size as any VDAC paralog. The one protein closest in size was conjugal transfer protein TraN, which is 28.5 kDa. However, only four of its residues exhibited sequence identity with the 10-amino acid VDAC immunogen query, which is smaller than a linear epitope. These results favor a model whereby VDAC1/VDAC3 associates with and is possibly incorporated by *O. tsutsugamushi* during infection. Lastly, the proposed metabolic competition hypothesis is based on correlative relationships between bacterial burden, metabolite depletion, and mitochondrial dysfunction and does not establish directionality or causality. Future studies employing proteomic analyses of *O. tsutsugamushi* membrane fractions, biochemical interaction assays, higher-resolution imaging approaches, and targeted metabolic flux analyses will be required to determine whether VDAC participates directly in host–pathogen exchange. Nevertheless, by demonstrating that *O. tsutsugamushi* infection perturbs mitochondrial function without inducing organelle loss and by identifying a selective association with VDAC, this work provides a novel framework for how obligate intracellular pathogens with limited metabolic capacity may engage mitochondrial-associated processes to support intracellular replication.

## Figures and Tables

**Table 1:** Sequence homology-based analyses assessing potential cross-reactivity between VDAC proteins and *O. tsutsugamushi*.

Query	Protein Description ^a^	Accession No. (Ikeda Locus Tag)	Theoretical Molecular Weight (kDa)	Amino Acid Identity with VDAC Sequence ^b^
VDAC1 (NP_003365.1)	NSS ^c^	–	31	–
VDAC2 (NP_001378892.1)	NSS	–	32	–
VDAC3 (NP_005653.3)	NSS	–	31	–
pan-VDAC [sc-390996] immunogen: YKREHINLGCDMDFDIAGPSIRGALVLGYEGWLAGYQMNF	NSS	–	–	–
pan-VDAC [sc-390996] immunogen residues 1–10 ^d^: YKREHINLGC	Penicillin-binding protein 2	WP_231840748.1 (OTT_RS07620)	62.9	4/10
	Hypothetical protein	WP_012461281 (OTT_RS03085)	80.3	5/10
	Pyrimidine dimer DNA glycosylase/endonuclease V	WP_012461702.1 (OTT_RS05700)	20.1	6/10
pan-VDAC [sc-390996] immunogen residues 11–20: DMDFDIAGPS	Polyribonucleotide nucleotidyltransferase	WP_012461866.1 (OTT_RS06590)	81.5	5/10
	Hypothetical protein	WP_012461372.1 (OTT_RS03650)	7.0	5/10
	NADH-quinone oxidoreductase subunit NuoF	WP_012461501.1 (OTT_RS04435)	46.4	4/10
pan-VDAC [sc-390996] immunogen residues 21–30: IRGALVLGYE	Cytochrome B	WP_012461337.1 (OTT_RS03475)	45.8	5/10
	Outer membrane protein assembly factor BamA	WP_012460754.1 (OTT_RS00155)	86.5	5/10
	Conjugal transfer protein TraN	BAG39515.1 (OTT_RS10350)	28.5	4/10
pan-VDAC [sc-390996] immunogen residues 31–40: GWLAGYQMNF	T4SS effector RisK1	WP_012462084.1 (OTT_RS07870)	71.1	3/10
	DUF2672 domain-containing protein	WP_012461064.1 (OTT_RS01805)	14.3	4/10
	Ester Cyclase	WP_050731349.1 (OTT_RS02645)	20.1	4/10
VDAC2 [PA5-28106] immunogen: residues 229–294	NSS	**-**	-	-
VDAC2 [PA5-28106] immunogen residues 229–245: RFGIAAKYQLDPTASIS	Ribonucleoside-diphosphate reductase subunit alpha	WP_012461949.1 (OTT_RS07050)	68.1	12/17
	Tetratricopeptide repeat protein	WP_012462244.1 (OTT_RS08855)	43.1	17/17
	Sensor histidine kinase	WP_012461111.1 (OTT_RS02090)	33.3	8/17
VDAC2 [PA5-28106] immunogen residues 246–262: AKVNNSSLIGVGYTQTL	NTP/NDP exchange transporter	WP_012461505.1 (OTT_RS04455)	57.3	21/17
	Trigger factor	WP_012461404.1 (OTT_RS03830)	55.4	7/17
	CNNM/CorC family transporter	WP_012461270.1 (OTT_RS03030)	47.1	7/17
VDAC2 [PA5-28106] immunogen residues 263–280: RPGVKLTLSALVDGKSI	50S ribosomal protein L19	WP_012460802.1 (OTT_RS04700)	14.3	7/17
	Ankyrin repeat domain-containing protein	WP_012461452.1 (OTT_RS04140)	53.7	16/17
	2-oxoglutarate dehydrogenase E1 component	WP_012461273.1 (OTT_RS03045)	109.2	14/17
VDAC2 [PA5-28106] Immunogen residues 281–294: NAGGHKVGLALELEA	Recombinase RecA	WP_012461603.1 (OTT_RS05040)	38.7	5/15
	Hypothetical protein	WP_012461135.1 (OTT_RS02240)	66.1	9/15
	ABC transporter ATP-binding protein/permease	WP_012461184.1 (OTT_RS02555)	66.8	7/15

^a^ Underlined results represent outer- or inner-membrane or periplasmic proteins. ^b^ Identities are based on both consecutive and non-consecutive amino acids. ^c^ NSS, no significant similarity. ^d^ Because the amino acid positions of this conserved sequence varies among the three VDAC isoforms, the residue numbers refer to the immunogen sequence itself.

## Data Availability

The original contributions presented in this study are included in the article. Further inquiries can be directed to the corresponding author.

## Abbreviations

The following abbreviations are used in this manuscript:

MTOC	Microtubule-organizing center
TCA	Tricarboxylic acid
VDAC	Voltage-dependent anion channel
PBS	Phosphate-buffered saline
MTT	3-(4,5-dimethylthiazol-2-yl)-2,5-diphenyltetrazolium bromide
MOI	Multiplicity of infection
hpi	Hours post-infection
DNA	Deoxyribonucleic acid
PCR	Polymerase chain reaction
qPCR	Quantitative polymerase chain reaction
RIPA	Radioimmunoprecipitation assay
EDTA	Ethylenediaminetetraacetic acid
SDS-PAGE	Sodium dodecyl sulfate–polyacrylamide gel electrophoresis
TBS	Tris-buffered saline
TSA56	56 kDa type-specific antigen
GAPDH	Glyceraldehyde-3-phosphate dehydrogenase
IgG	Immunoglobulin G
PFA	Paraformaldehyde
BSA	Bovine serum albumin
DAPI	4′,6-diamidino-2-phenylindole
TEM	Transmission electron microscopy
SIM	Structured illumination microscopy
STORM	Stochastic optical reconstruction microscopy
ANOVA	One-way analysis of variance
BLAST	Basic Local Alignment Search Tool
NSS	No significant similarity
PRDX3	Peroxiredoxin 3
TOM20	translocase of outer mitochondrial membrane (TOM) complex
OMP	Outer membrane protein
WCL	Whole cell lysate
ATP	Adenosine triphosphate
ER	Endoplasmic reticulum
ERAD	ER-associated degradation
MAMs	Mitochondria-associated membranes
